# Diffusion MRI Markers of Symptom Interference and Survival in Head and Neck Cancer Survivors

**DOI:** 10.3390/brainsci16101045

**Published:** 2026-09-29

**Authors:** Sahil Bajaj, Christopher Chad Quarles, Katherine Gilmore, Ying Qiao, Chinmay Mokashi, Max Wintermark, Jeffrey S. Wefel, Kyle R. Noll, Michelle Zalles, Michael Roth

**Affiliations:** 1 Department of Cancer Systems Imaging, Division of Diagnostic Imaging, The University of Texas MD Anderson Cancer Center, Houston, TX 77054, USA; 2 Cancer Neuroimaging Research Program, The University of Texas MD Anderson Cancer Center, Houston, TX 77030, USA; 3 Cancer Neuroscience Program, The University of Texas MD Anderson Cancer Center, Houston, TX 77030, USA; 4 Cancer Survivorship Program, The University of Texas MD Anderson Cancer Center, Houston, TX 77030, USA; 5 Department of Radiology, The University of Texas Medical Branch (UTMB), Galveston, TX 77555, USA; 6 Department of Neuro-Oncology, Division of Cancer Medicine, The University of Texas MD Anderson Cancer Center, Houston, TX 77030, USA; 7 Department of Pediatrics, Division of Pediatrics, The University of Texas MD Anderson Cancer Center, Houston, TX 77030, USA

**Keywords:** quality of life, gray matter, white matter, diffusion tensor imaging, survivorship, glymphatic function

## Abstract

**Highlights:**

**What are the main findings?**

- Regional gray and white matter microstructure were associated with survival and symptom interference in long-term HN cancer survivors.
- DTI-ALPS modified region-specific associations between brain microstructure, survival, and symptom interference.

**What are the implications of the main findings?**

- Findings support a region-specific brain resilience framework in long-term cancer survivorship.
- dMRI-based microstructural and glymphatic markers may provide noninvasive biomarkers of brain health and survivorship outcomes.

**Abstract:**

**Background:** Diffusion MRI (dMRI) provides sensitive markers of brain microstructure and glymphatic function. The interplay between brain microstructure and glymphatic function in shaping long-term survivorship outcomes remains unclear. We examined associations of dMRI markers with survival and symptom interference in long-term head and neck (HN) cancer survivors and tested whether glymphatic function modified these associations. **Methods:** dMRI data from 135 long-term HN cancer survivors were analyzed. Radial diffusivity (RD) and fractional anisotropy (FA) were quantified across 76 gray matter regions and 45 white matter tracts. Glymphatic function was indirectly estimated using diffusion tensor imaging analysis along the perivascular space (DTI-ALPS) index. Survival status was determined through clinical follow-up, and symptom interference was assessed using the MD Anderson Symptom Inventory. Stepwise regression identified microstructural measures associated with survival and symptom interference, followed by moderation analyses testing the role of DTI-ALPS. **Results:** Reduced RD in the left medial orbitofrontal cortex (MOFC), higher FA in the left and lower FA in the right entorhinal cortex, and higher FA in the right extreme capsule were associated with longer survival (*p <* 0.0006, corrected for multiple comparisons). Lower RD in the left uncinate fasciculus (UF) and superior temporal cortex (STC) and lower FA in the right caudal anterior cingulate cortex (CACC) were associated with lower symptom interference (*p <* 0.0006, corrected for multiple comparisons). Higher DTI-ALPS strengthened the association between preserved MOFC microstructure and longer survival (β = −1.95, *p <* 0.001) and moderated the associations of microstructure in the UF (βs > 4.08, *p <* 0.001), STC (β = 5.82, *p <* 0.001), and CACC (β = 16.47, *p <* 0.001) with symptom interference. **Conclusions:** dMRI markers of brain microstructure and glymphatic function are associated with long-term outcomes in HN cancer survivors. These findings support a region-specific resilience framework and highlight the potential of dMRI-based biomarkers for characterizing brain health in cancer survivorship.

## 1. Introduction

Brain health in cancer survivors reflects a delicate balance between neural vulnerability and resilience mechanisms. Long-term cancer survivorship increasingly depends not only on disease control but also on the preservation of brain health, cognitive capacity, and quality of life [1,2]. Head and neck (HN) cancers, in particular, are associated with substantial long-term neurocognitive and psychosocial challenges due to the neurotoxic effects of multimodal cancer treatments such as chemotherapy, radiation therapy, and surgical interventions [3,4,5]. These treatments can induce widespread neurobiological alterations that extend beyond focal lesions, affecting distributed gray matter (GM) and white matter (WM) networks critical for emotion regulation, motivation, and everyday functioning [6,7]. Despite improved survival rates in this population, the neural mechanisms that differentiate vulnerable from resilient brain trajectories in HN cancer survivors remain poorly understood.

Two of the most informative diffusion magnetic resonance imaging (dMRI)-derived metrics–radial diffusivity (RD) and fractional anisotropy (FA)–reflect complementary aspects of tissue microstructure and provide a multidimensional view of microstructural health across both GM regions and WM tracts with well-characterized diffusion anisotropy [8]. Cancer-related neurotoxicity and systemic inflammation can disrupt these diffusion metrics. Prior neuroimaging work in cancer survivors has documented alteration in RD and FA in distributed GM (including frontotemporal, limbic, and subcortical regions) and WM (including corpus callosum and longitudinal fasciculus) regions, patterns consistent with demyelination, axonal injury, or altered extracellular fluid dynamics [9,10]. However, these alterations are often heterogeneous across individuals, suggesting that additional resilience mechanisms—possibly related to fluid clearance and metabolic homeostasis—may modulate the neurobiological and behavioral consequences of cancer and its treatments.

A growing body of evidence highlights the glymphatic function as a critical contributor to brain health and repair [11,12]. Although the brain parenchyma lacks conventional lymphatic vessels, glymphatic function facilitates cerebrospinal fluid (CSF) and interstitial fluid (ISF) exchange through perivascular pathways, with CSF entering along periarterial spaces to support fluid and solute clearance [13,14]. While exogenous contrast agents can assess glymphatic function, concerns regarding invasiveness and potential toxicity have prompted the development of noninvasive alternatives, such as the diffusion tensor imaging along perivascular spaces (DTI-ALPS) index. Recent advances in dMRI have enabled the indirect quantification of glymphatic function through the DTI-ALPS index [15], which captures diffusion anisotropy along perivascular pathways. Reduced ALPS values have been associated with impaired waste clearance and neurodegenerative processes, while higher values indicate more efficient interstitial fluid exchange [16,17]. Despite emerging evidence linking glymphatic function to cognitive and sleep health, its contribution to brain integrity and behavioral resilience in cancer survivorship has not yet been systematically investigated.

Both GM and WM structures play distinct yet complementary roles in shaping survivorship outcomes. The medial orbitofrontal cortex (MOFC) and entorhinal cortex (ENT) regions are, in particular, essential for affective regulation, reward processing, and memory integration [18,19,20,21,22,23,24]. Similarly, other temporal and limbic structures such as the superior temporal cortex (STC), anterior cingulate cortex (ACC), and caudate nucleus contribute to emotional well-being, fatigue, and motivational drive [22,25,26,27,28]—domains often disrupted in HN cancer survivors [4,29,30]. WM tracts such as the uncinate fasciculus (UF), which interconnects frontal and temporal regions, also mediate emotion-related and executive processes [31,32]. The inferior longitudinal fasciculus (ILF) is another key WM tract connecting the occipital and temporal lobes, involved in visual processing, memory, and emotion [33,34,35]. While it does not directly determine survival, its integrity contributes to cognitive and affective functioning [34], which can support brain resilience and indirectly influence survival-related outcomes and overall well-being [34,36]. In contrast, the extreme capsule (EXT CAP)—a major associative pathway connecting frontal, temporal, and insular cortices—plays a more integrative role in higher-order cognitive control, language, and emotion regulation [37,38]. Integrity of the EXT CAP has been linked to preserved executive and affective functioning [37,39], which are critical for maintaining functional capacity and adaptive responses to disease burden. Together, these findings indicate that integrity of specific GM regions and WM pathways supports neural health and underlies resilience-related mechanisms relevant to long-term outcomes. These regions likely serve as neural substrates through which microstructural integrity relates to both survival and symptom burden in this population. Joint evaluation of RD and FA across GM and WM captures complementary markers of microstructural vulnerability and resilience. Importantly, integrating these diffusion metrics with DTI-ALPS index provides a mechanistic framework for understanding how brain clearance and maintenance processes may moderate the relationship between regional tissue integrity and survival-related and functional outcomes.

In cancer survivorship, symptom interference—defined as the extent to which cancer and treatment-related symptoms disrupt daily life—offers a clinically meaningful measure of patient well-being. Symptom interference can be broadly categorized into activity-related domains (e.g., interference with work, walking, and general activity) and affective domains (e.g., mood, relationships, enjoyment of life) [40]. The present study tested whether preserved microstructural integrity within cortical GM, subcortical GM, and WM fiber bundles was associated with longer survival and lower symptom interference, and whether ALPS index would strengthen these associations. We hypothesized that: (a) microstructural biomarkers of survival—specifically, reduced RD and elevated FA within key cortical (e.g., MOFC, ENT and ACC) and subcortical regions (e.g., caudate nucleus)—would be associated with longer survival and reduced affective interference; (b) microstructural WM biomarkers of daily functioning—characterized by reduced RD and increased FA in emotion-regulatory circuits involving the UF, ILF and EXT CAP—would be associated with lower activity-related and affective symptom interference; and (c) higher DTI-ALPS values would strengthen these brain–behavior associations, enhancing the survival benefits of preserved neuronal integrity and amplifying the resilience effects of reduced symptom interference associated with preserved GM regions and WM pathways. In particular, by examining both microstructural and glymphatic function-related mechanisms in a well-characterized cohort of long-term HN cancer survivors, this study seeks to bridge two traditionally separate domains of neuro-oncology: structural integrity and markers of systemic clearance function.

## 2. Materials and Methods

### 2.1. Participant Characteristics

A total of 135 long-term HN cancer survivors (mean age at MRI scan = 66.04 years, SD = 11.83; 107 males, 28 females) were included in the present study (see Table 1 for patient characteristics). Long-term survivorship reflected a broad range of post-diagnosis follow-up, with MRI scans obtained 0.04–29.73 years (median = 5.22 years) after initial diagnosis (independent of cancer type) and MDASI symptom assessments collected 2.14–27.62 years (median = 6.73 years) post-diagnosis; diagnosis dates were unavailable for six patients. Inclusion criteria required a confirmed history of HN cancer, completion of primary cancer treatment, and no current evidence of recurrent or metastatic disease. Exclusion criteria included a history of neurological or psychiatric conditions unrelated to cancer, prior traumatic brain injury, or contraindications to MRI. All participants were in the survivorship phase of care at the time of MRI acquisition.

Tumor/cancer type and primary tumor locations were heterogeneous across the cohort; Table 2 depicts the distribution of patients across tumor/cancer type and tumor location categories. Disease stage at diagnosis was distributed as follows: 25 participants had Stage I disease, 19 had Stage II, 23 had Stage III, 46 had Stage IV, and 6 had an unknown stage. Clinical data, including demographic characteristics, cancer diagnosis, and follow-up survival status, were obtained from institutional medical records. Survival status was determined through extended clinical follow-up and categorized as alive (*n* = 86), deceased (*n* = 46), or unavailable (*n* = 3). Last contact dates were unavailable for 33 of the remaining 132 patients. Consequently, survival duration since initial diagnosis could be calculated for 99 patients, with a mean (SD) survival time of 15.41 (5.54) years for those alive (*n* = 67) and 14.06 (5.88) years for those deceased (*n* = 32). The specific cause of death for the deceased participants was not available, but it could potentially include cancer recurrence or second primary malignancies, treatment-related late effects (e.g., cardiovascular or pulmonary complications), infections, nutritional or metabolic complications, or other comorbid conditions, reflecting the multifactorial risks faced by the survivors. This study was conducted under protocols approved by the Institutional Review Board (IRB) at The University of Texas MD Anderson Cancer Center.

### 2.2. Cancer Treatment Regimens

Among the 135 patients, the most common treatment regimen was chemotherapy combined with radiation therapy (CHEMO + XRT; 29 patients, 21.5%), followed by surgery alone (24 patients, 17.8%), surgery with radiation (SURGERY + XRT; 16 patients, 11.9%), and radiation therapy alone (XRT; 14 patients, 10.4%). Trimodality treatment consisting of surgery, chemotherapy, and radiation was administered to 10 patients (7.4%), and chemotherapy combined with immunotherapy and radiation (CHEMO + IMMUNO + XRT) was given to 8 patients (5.9%). Other treatments included chemotherapy with surgery (5 patients, 3.7%), chemotherapy alone (5 patients, 3.7%), other surgical interventions (OTHSURG; 4 patients, 3.0%), chemotherapy with immunotherapy (3 patients, 2.2%), immunotherapy with radiation (2 patients, 1.5%), and no documented treatment (2 patients, 1.5%). Rare combinations, such as chemotherapy with endocrine therapy, chemotherapy with proton therapy, surgery with hyperthermia, endocrine therapy with surgery, and other complex multimodal regimens, were each observed in 1 patient (0.7% each).

### 2.3. Symptom Interference Assessment

Symptom interference was assessed using the MD Anderson Symptom Inventory (MDASI), a validated self-report measure of symptom burden in cancer populations [40]. Participants rated the degree to which cancer-related symptoms interfered with different aspects of daily functioning on a scale from 0 (“did not interfere”) to 10 (“interfered completely”). To capture distinct dimensions of real-world functioning, interference items were grouped into two empirically derived subscales: (a) Activity-related interference reflecting disruption to physical and routine activities (i.e., general activity, walking, work) and (b) Affective interference reflecting disruption to emotional and social well-being (i.e., mood, relationships with others, enjoyment of life).

### 2.4. MRI Acquisition

All MRI data were acquired on a 3 Tesla GE Signa scanner using a 24-channel head coil. dMRI sequences were collected with the following parameters: repetition time (TR) = 8000 ms, echo time (TE) = 70 ms, flip angle = 90°, voxel size = 0.9 × 0.9 × 5.0 mm^3^, and 20 or 27 diffusion directions (b = 1000 or 1200 s/mm^2^), with 1 to 3 non-diffusion-weighted (b0) volumes. Quality assurance included visual inspection for motion and distortion artifacts and automated checks for signal dropout.

dMRI data analyzed in this study were derived from clinical scans of 321 HN cancer patients, with acquisition parameters varying due to differences in clinical protocols and scanner settings. To address this heterogeneity, rigorous quality control (QC) procedures were applied. Only datasets with 20 or 27 diffusion-weighted directions were included, as these provided sufficient angular coverage for robust reconstruction and microstructural quantification. Therefore, participants were excluded if they had datasets with fewer than 20 or more than 27 diffusion directions, as these either lacked adequate sampling for accurate diffusion modeling or were inconsistent with the majority of included scans. This approach balances the inclusion of clinically relevant data with stringent QC criteria, ensuring that downstream analyses reflect high-quality and comparable diffusion measurements across participants.

### 2.5. Diffusion MRI Preprocessing and Tractography

All dMRI data and tractography were processed using the DSI Studio (Hou version) pipelines [41], beginning with the generation of SRC reconstruction files from the NIFTI images. A rigorous QC procedure was applied to ensure the integrity and suitability of the SRC files. QC included verification of image dimensions, spatial resolution, the number of diffusion-weighted images, and shell counts, as incomplete scans or varying DWI counts may indicate potential acquisition issues. Additionally, DSI Studio evaluates datasets for low-quality outliers by computing the neighboring DWI correlation, a metric sensitive to artifacts such as eddy currents, head motion, or hardware-related issues that can compromise signal quality. Datasets exhibiting a neighboring DWI correlation below 0.7 underwent manual inspection of raw DWI images to determine whether artifacts could be corrected; datasets failing this evaluation were excluded from further analyses.

All dMRI data were bias-field corrected using the *b*0 image and reconstructed in standard MNI space using q-space diffeomorphic reconstruction (QSDR) implemented in DSI Studio [42]. QSDR generates voxel wise spin distribution functions (SDFs) [43], enabling high-resolution mapping of diffusion properties within both cortical and subcortical GM and WM and in a common anatomical space. A diffusion sampling length ratio of 1.25 was used, and the output resolution was 2 mm isotropic. Conventional diffusion metrics, including RD and FA, were derived and primarily reflect WM microstructural integrity; however, QSDR additionally enables quantification of regional microstructural organization within GM. Restricted diffusion was quantified using restricted diffusion imaging [44].

Deterministic whole-brain fiber tracking was performed using DSI Studio’s command-line interface based on the SDF reconstruction [45], with augmented tracking strategies to improve reproducibility [46]. Unless otherwise specified, default tractography parameters were used as defined by the DSI Studio documentation. These include automated seed and tract counts determined by the seed-to-voxel ratio, Otsu-based anisotropy thresholding with randomized selection between 0.5 and 0.7 of the threshold, randomized angular thresholds between 45° and 90°, step size set to voxel spacing, zero smoothing, dataset-specific minimum and maximum tract lengths, and a default random seed of zero. Tracks shorter than 30 mm or longer than 200 mm were discarded.

### 2.6. Region-of-Interest (ROI) and WM Fiber Bundles of Interest

To comprehensively characterize microstructural integrity, RD and FA values were extracted from 62 cortical GM regions defined by the Desikan–Killiany atlas [47], 14 main subcortical GM regions defined by ASEG atlas [48], and 45 major WM fiber bundles defined by the HCP842 atlas [49]. Here, the Desikan–Killiany and ASEG atlases were selected to provide standardized, widely used anatomical parcellations of cortical and subcortical gray matter, respectively [47,50]. The HCP842 atlas was used for white matter analysis because it provides a standardized parcellation of major WM tracts optimized for dMRI and tractography-based analyses [49,51]. See Table 3 for the list of GM regions and WM fiber bundles included in the analyses. Mean RD and FA values were computed for each GM ROI and for each WM fiber bundle using each participant’s registered maps.

### 2.7. Quantification of Glymphatic Function (DTI-ALPS Index)

Glymphatic function was indirectly assessed using the DTI-ALPS index [15,52]. The DTI-ALPS index was computed following established procedures [15,52]. Briefly, diffusivity values along x-, y-, and z-axes were extracted from ROIs adjacent to the lateral ventricles—specifically, projection and association fiber areas aligned with perivascular spaces. The ALPS index was calculated as:

$$\mathrm{ALPS}\;\mathrm{index}\;=\;\frac{D_{x,\mathrm{projection}}\;+\;D_{x,\mathrm{association}}}{D_{y,\mathrm{projection}}\;+\;D_{z,\mathrm{association}}}$$

where $D_{x}$, $D_{y}$, and $D_{z}$ represent diffusivities along respective axes. All ROI placements and ALPS calculations were visually inspected to ensure anatomical accuracy and data quality.

### 2.8. Statistical Analyses

#### 2.8.1. Stepwise Regression Models

Stepwise multiple regression analyses were conducted to identify regional and WM path specific RD and FA measures associated with (1) survival status (as binary outcome) and (2) symptom interference (activity-related and affective domains). Age and sex were included as standard covariates in all the analyses, and additionally, the time gap in days between the MRI scan and MDASI (activity-related and affective) data collection (for symptom interference analyses only) was included as a covariate. This approach allowed the model to account for potential variability in outcome measures related to timing of data collection while allowing the stepwise procedure to evaluate its relative contribution alongside RD and FA measures. Separate analyses were performed for GM regions and WM pathways, and RD and FA were analyzed in distinct models. All the measures and covariates were entered stepwise using an inclusion criterion of *p* < 0.05.

#### 2.8.2. Moderation Analyses

Moderation effects of glymphatic function were tested using PROCESS (v4.2) in SPSS 29.0.245. Each significant RD and FA measure from the stepwise analysis was entered as an independent variable, the ALPS index as a moderator, and the relevant outcome (survival or symptom interference) as the dependent variable. Age and sex were included as standard covariates in all models. Additionally, the time gap in days between the MRI scan and MDASI (activity-related and affective) data collection (for symptom interference analyses only) was included as an additional covariate. All continuous variables were mean centered prior to computing interaction terms to reduce multicollinearity between main effects and interaction terms and to facilitate the interpretability of regression coefficients. Significant moderation was inferred when the interaction term (Microstructure [RD or FA] × ALPS) reached *p* < 0.05. Simple slope analyses and interaction plots were used to visualize moderation effects.

#### 2.8.3. Multiple Comparison Correction

To reduce the risk of Type I error arising from the large number of neuroanatomical comparisons, Bonferroni correction was applied separately within the two prespecified anatomical families: GM and WM. For GM, 76 cortical and subcortical regions were evaluated for associations between diffusion measures (RD and FA) and the prespecified clinical outcomes. Accordingly, the Bonferroni-corrected significance threshold was *p* < 0.05/76 = 0.00066. For WM, 45 major pathways were evaluated, yielding a corrected threshold of *p* < 0.05/45 = 0.00111. These thresholds were applied to the corresponding regional/pathway regression analyses for both RD and FA.

GM and WM were treated as separate hypothesis families because they represent anatomically distinct tissue compartments with different microstructural organization and biological interpretation. RD and FA were retained within each tissue-specific family because they represent complementary diffusion-derived measures of the same anatomical structures rather than independent anatomical hypothesis sets.

The correction was therefore designed to control the family-wise error rate at 5% across the 76 regional comparisons within the GM family and across the 45 pathway comparisons within the WM family. The different clinical outcomes were considered distinct, prespecified outcome domains addressing different aspects of survivorship (survival, activity-related interference, and affective interference), rather than being pooled into a single undifferentiated outcome family.

ALPS moderation analyses were performed only for regional/pathway associations that survived the corresponding Bonferroni correction. These interaction analyses were therefore conducted as follow-up tests of corrected associations rather than as an additional exploratory screen across all anatomical regions and pathways.

### 2.9. Exploratory ALPS Analyses

To provide context for interpreting moderation results, ALPS was first compared between survival groups (alive vs. deceased) using an independent-samples *t*-test. Subsequently, Spearman rank-order correlations were calculated between the moderating variable ALPS and key outcome measures, including symptom interference severity and microstructural metrics (i.e., RD and FA) of the identified GM regions and WM pathways that showed significant associations (via stepwise regression analysis) with survival and symptom interference. These exploratory analyses were intended to clarify the direction and strength of associations underlying the observed moderation effects.

## 3. Results

### 3.1. Participant Characteristics

Demographic and clinical characteristics of the HN cancer survivor cohort (*N* = 135) are summarized in Table 1. Participants had a mean age of 66.04 years (SD = 11.83) at MDASI data collection and were predominantly male (79.2%). At follow-up, 63.7% of survivors were alive, 34.1% were deceased, and survival data were missing for 2.2% (i.e., for 3 participants). All 135 participants had available brain MRI and symptom burden assessments; therefore, symptom-interference analyses were conducted in the full cohort (*N* = 135), whereas survival analyses were conducted in the 132 participants with available survival data. The “Alive” and “Deceased” subgroups were generally comparable with respect to age at MDASI data collection, age at last known survival status and initial diagnosis, sex, symptom interference scores, and the interval between MRI scanning and MDASI data collection but differed in age at the time of MRI scanning. Diagnosis dates were unavailable for 6 participants but were not required for any study analyses. Descriptive statistics (means and standard deviations [SD]) and results of between-group comparisons for these variables are summarized in Table 1.

### 3.2. Survival and Interference

The final regression equations (via Stepwise linear regression) between brain microstructure and both survival and interference were interpreted at *p* < 0.05. However, only the robust associations (i.e., the associations that survived multiple comparison correction) between brain microstructure and both survival and interference were reported and interpreted.

#### 3.2.1. Survival

**Figure 1:**
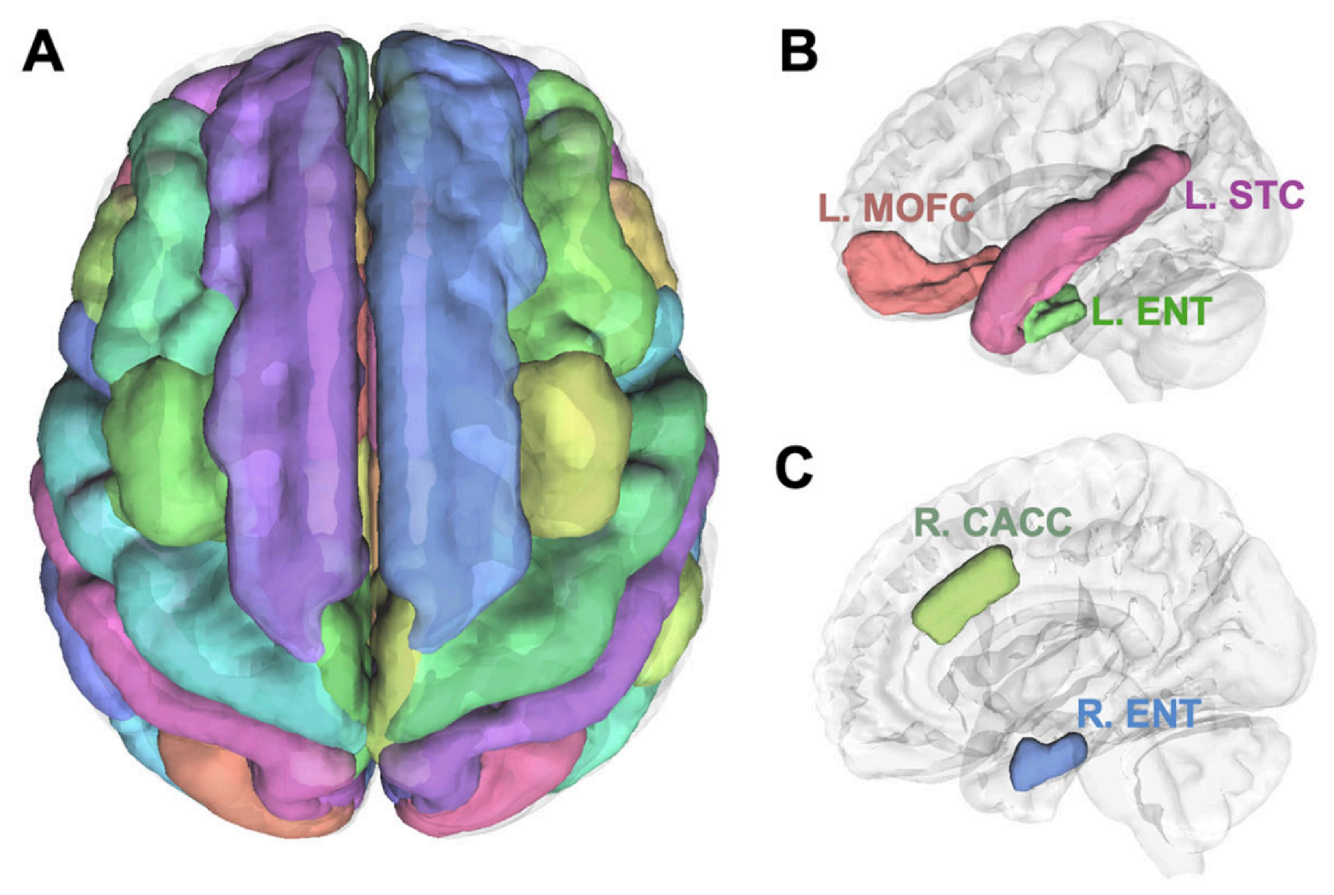
Anatomical distribution of the (**A**) 62 cortical brain regions, and (**B**,**C**) five identified regions that showed a significant association with survival and/or symptom interference. These regions are shown on a standard glass brain in MNI space after overlaying the Desikan–Killiany atlas.

**Figure 2:**
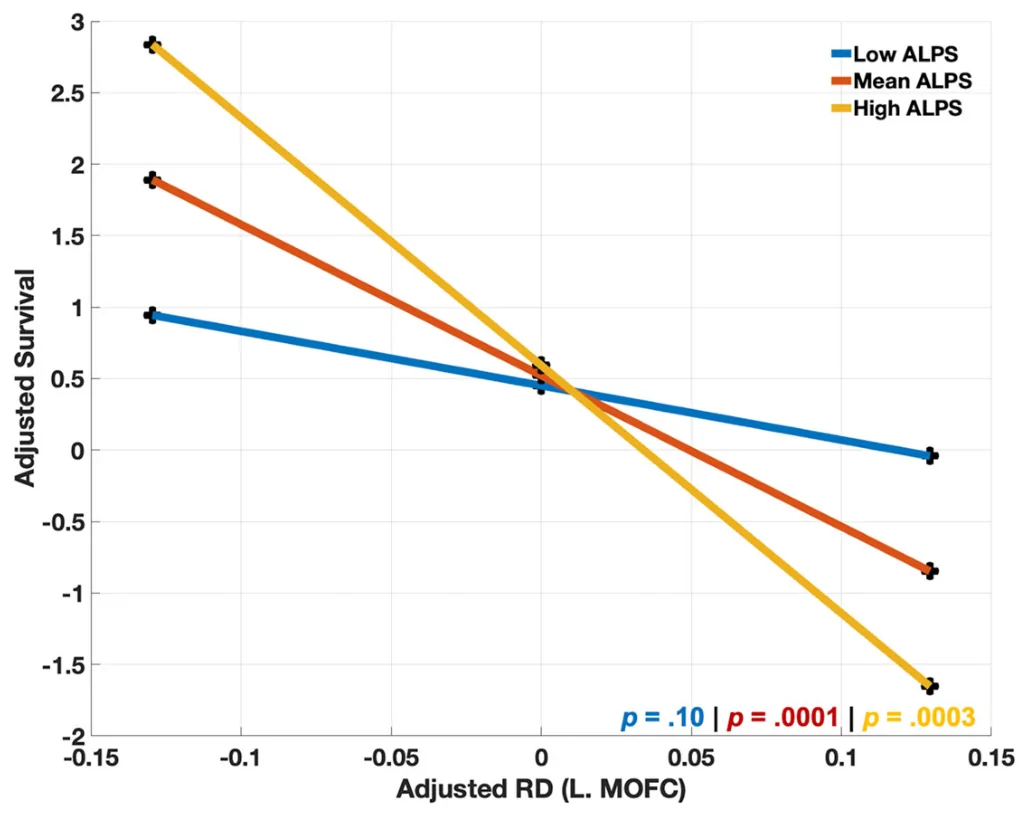
Moderation results: Role of ALPS in moderating the association between RD for MOFC and survival.

***RD for GM.*** The final model for RD (for GM) was significant, explaining a substantial proportion of variance in survival (R^2^ = 0.16, F-change = 4.86, *p* = 0.03). Here, lower RD in the left MOFC, moderated by higher ALPS values (i.e., ≥−0.11), was robustly associated with increased survival (β = −1.95, *p* < 0.001). Figure 1A shows the anatomical distribution of the 62 cortical brain regions visualized on a standard glass brain. Anatomical locations of identified regions are shown in Figure 1B and corresponding moderation results are plotted in Figure 2.

***FA for GM.*** The final model for FA (for GM) was significant, explaining a substantial proportion of variance in survival (R^2^ = 0.25, F-change = 6.77, *p* = 0.01). Here, elevated FA in the left ENT (β = 9.37, *p* < 0.001) and reduced FA in the right ENT (β = −7.29, *p* < 0.001) were robustly associated with increased survival, though these effects were not moderated by ALPS (ps > 0.05). Anatomical locations of identified regions are shown in Figure 1B,C.

***RD for WM.*** The final model for RD (for WM) was significant, explaining a modest proportion of variance in survival (R^2^ = 0.07, F-change = 9.99, *p* = 0.002). However, none of the WM pathways were robustly associated with survival.

**Figure 3:**
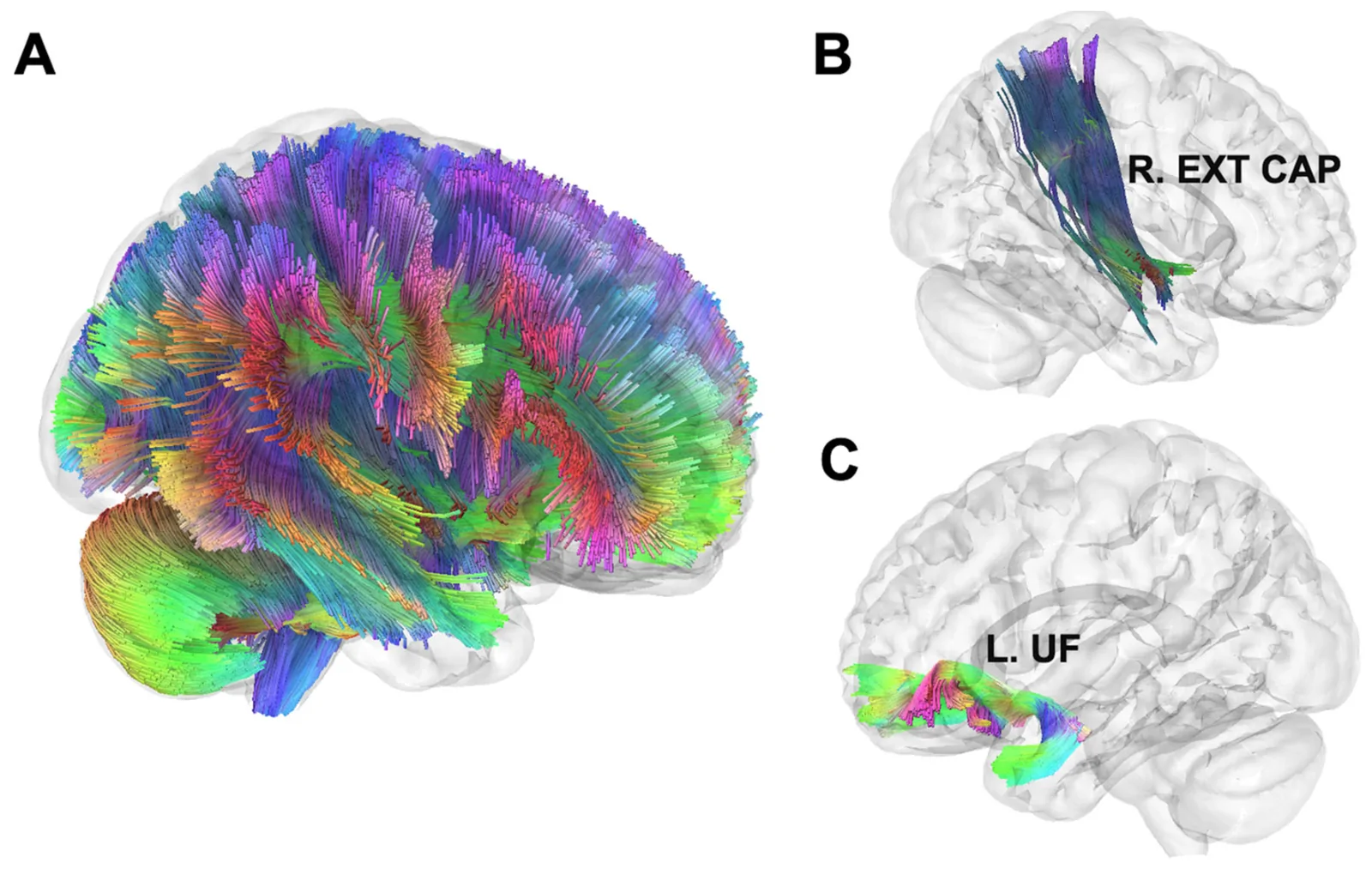
Distribution of (**A**) whole-brain WM pathways, and (**B**,**C**) two identified WM pathways that showed a significant association with survival and/or symptom interference. The displayed pathways are averaged across all participants.

***FA for WM.*** The final model for FA (for WM) was significant, explaining a modest proportion of variance in survival (R^2^ = 0.09, F-change = 4.96, *p* = 0.03). Here, elevated FA in the left EXT CAP was robustly associated with increased survival (β = 5.04, *p* < 0.001), though this effect was not moderated by ALPS. Figure 3A presents whole-brain WM tractography pathways averaged across all participants. Anatomical location of identified WM pathway is shown in Figure 3B.

#### 3.2.2. Activity-Related Interference

***RD for GM.*** The final model for RD (for GM) was significant, explaining a substantial proportion of variance in activity-related interference (R^2^ = 0.24, F-change = 4.00, *p* = 0.05). However, none of the GM regions were robustly associated with activity-related interference.

***FA for GM.*** The final model for FA (for GM) was significant, explaining a substantial proportion of variance in activity-related interference (R^2^ = 0.12, F-change = 6.76, *p* = 0.01). However, none of the GM regions were robustly associated with activity-related interference.

**Figure 4:**
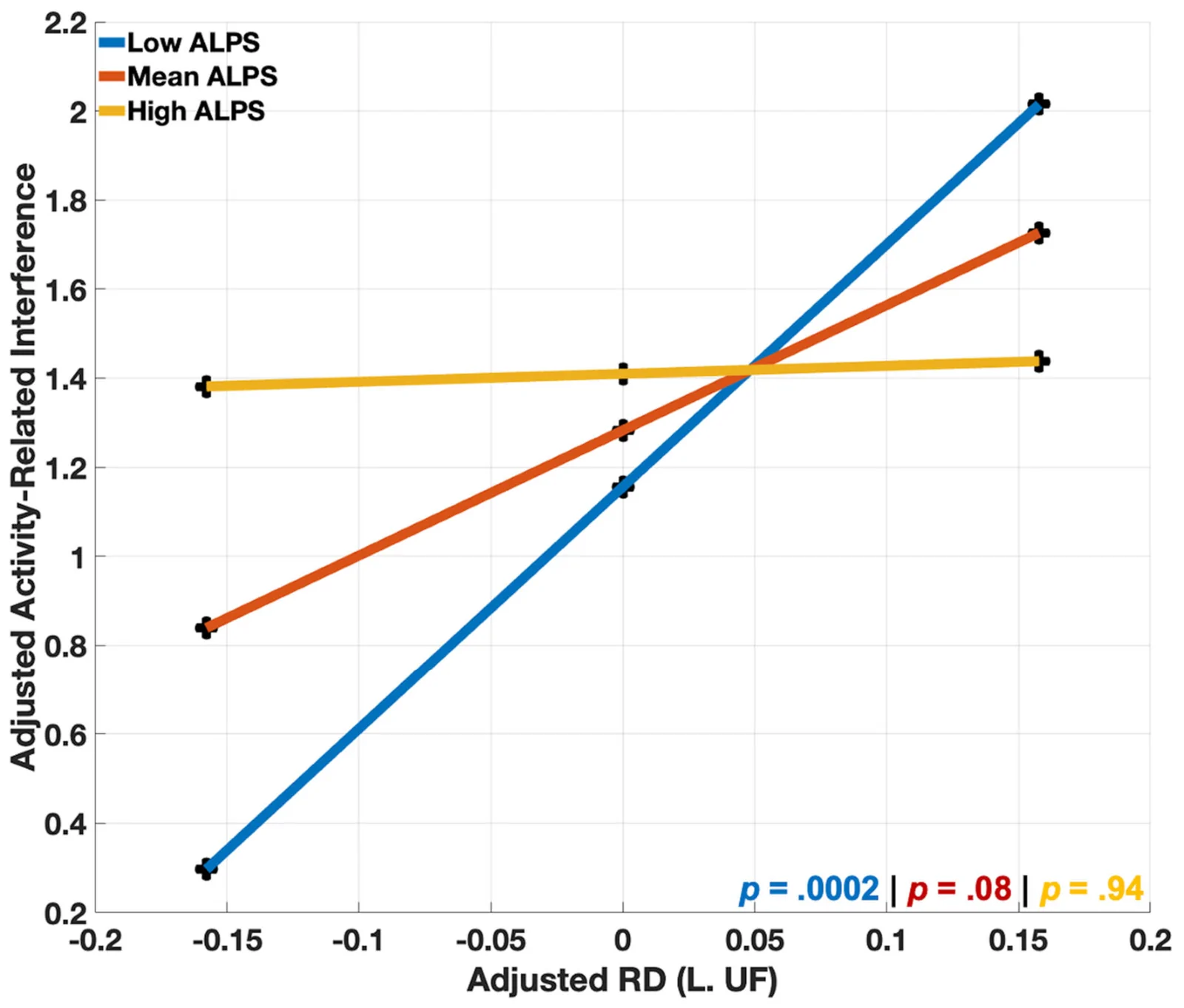
Moderation results: Role of ALPS in moderating the association between RD for UF and activity-related interference.

***RD for WM***. The final model for RD (for WM) was significant, explaining a substantial proportion of variance in activity-related interference (R^2^ = 0.18, F-change = 4.68, *p* = 0.03). Here, lower RD in the left UF, moderated by lower ALPS values (i.e., at ≤−0.01), was robustly associated with reduced activity-related interference (β = 6.61, *p* < 0.001). The anatomical location of the identified WM pathway is shown in Figure 3C and corresponding moderation results are plotted in Figure 4.

***FA for WM.*** The final model for FA (for WM) was significant, explaining a modest proportion of variance in activity-related interference (R^2^ = 0.05, F-change = 6.51, *p* = 0.01). However, none of the WM pathways were robustly associated with activity-related interference.

#### 3.2.3. Affective Interference

**Figure 5:**
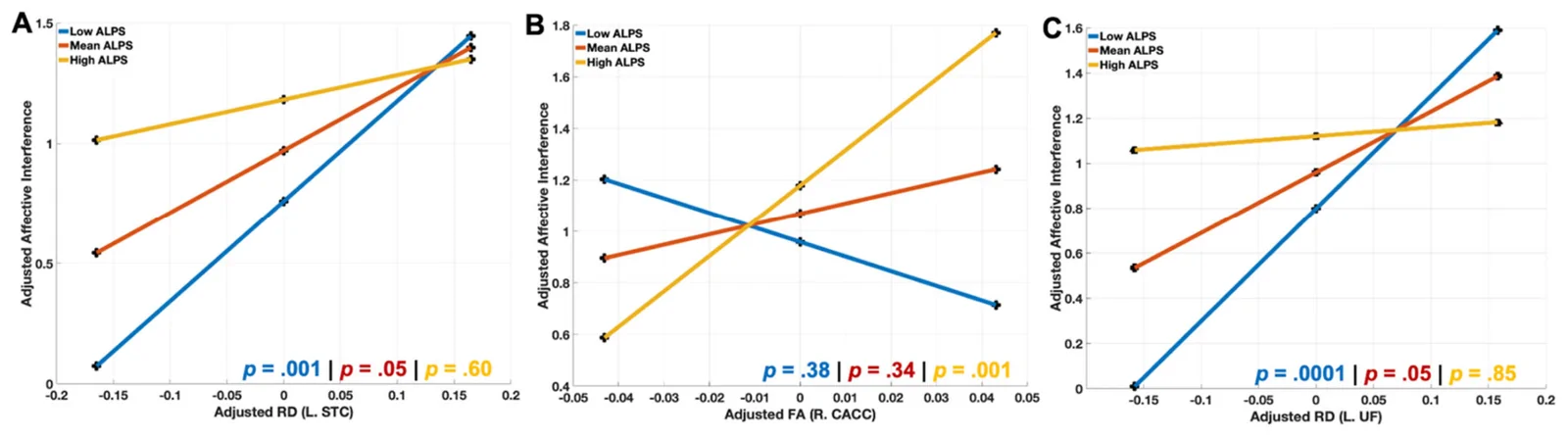
Moderation results: Role of ALPS in moderating the association between (**A**) RD for STC and affective interference; (**B**) FA for CACC and affective interference; and (**C**) RD for UF and affective interference.

***RD for GM.*** The final model for RD (for GM) was significant, explaining a substantial proportion of variance in affective interference (R^2^ = 0.19, F-change = 4.66, *p* = 0.03). Here, lower RD in the left STC, moderated by lower ALPS values (i.e., at ≤−0.01), was robustly associated with reduced affective interference (β = 5.82, *p* < 0.001). Anatomical location of identified region is shown in Figure 1B and corresponding moderation results are plotted in Figure 5A.

***FA for GM.*** The final model for FA (for GM) was significant, explaining a substantial proportion of variance in affective interference (R^2^ = 0.21, F-change = 4.23, *p* = 0.04). Here, lower FA in the right CACC was robustly associated with reduced affective interference (β = 16.47, *p* < 0.001) when ALPS was high (i.e., ≥0.04), but with greater affective interference only when ALPS was very low (i.e., ≤−0.32). Anatomical location of identified region is shown in Figure 1C and corresponding moderation results are plotted in Figure 5B.

***RD for WM***. The final model for RD (for WM) was significant, explaining a substantial proportion of variance in affective interference (R^2^ = 0.16, F-change = 4.14, *p* = 0.04). Here, lower RD in the left UF, moderated by lower ALPS values (i.e., at ≤0.003), was robustly associated with reduced affective interference (β = 4.08, *p* < 0.001). The anatomical location of the identified WM pathway is shown in Figure 3C and corresponding moderation results are plotted in Figure 5C.

***FA for WM.*** The final model for FA (for WM) was significant, explaining a modest proportion of variance in affective interference (R^2^ = 0.06, F-change = 9.14, *p* = 0.003). However, none of the WM pathways were robustly associated with affective interference.

All results, including measures that survived multiple-comparison correction and ALPS moderation effects, with corresponding β coefficients, interaction effects, and *p*-values, are summarized in Table 4.

### 3.3. Exploratory Associations Between ALPS and Outcome Measures

**Figure 6:**
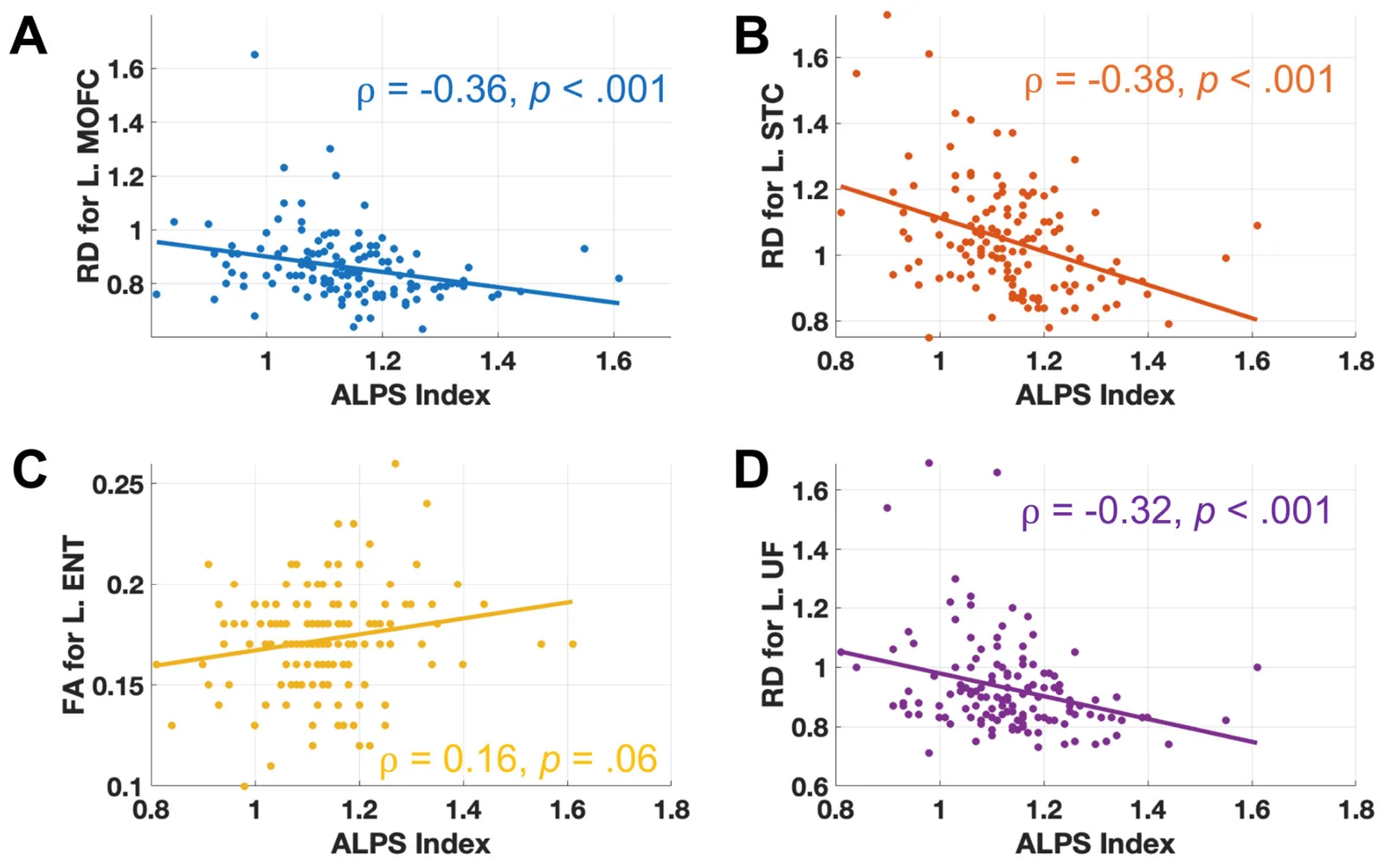
Association between ALPS index and (**A**) RD for MOFC; (**B**) RD for STC; (**C**) FA for ENT; and (**D**) RD for UF.

An independent-sample *t*-tests revealed that ALPS index did not differ significantly between the Alive and Deceased groups (*t*(130) = −1.54, *p* = 0.12). Spearman correlation analyses revealed significant associations between the moderating variable ALPS and several key outcome measures. ALPS index was not associated with symptom interference severity (activity-related interference: ρ = −0.06, *p* = 0.50; affective interference: ρ = 0.05, *p* = 0.54). However, higher ALPS index was significantly correlated with lower RD and greater FA in specific GM regions (RD for left MOFC: ρ = −0.36, *p* < 0.001 [Figure 6A], RD for left STC: ρ = −0.38, *p* < 0.001 [Figure 6B], and FA for left ENT: ρ = 0.16, *p* = 0.06 [Figure 6C]) and specific WM pathways (RD for left UF: ρ = −0.32, *p* < 0.001 [Figure 6D]). No significant associations were found between ALPS and FA for right ENT (ρ = 0.02, *p* = 0.81), right CACC (ρ = 0.02, *p* = 0.82), and right EXT CAP (ρ = 0.10, *p* = 0.23). These exploratory results suggest that greater glymphatic function, as indexed by ALPS, is associated with preserved WM microstructural integrity, as reflected by lower RD and higher FA, thereby providing context for the moderation effects observed in the primary analyses.

## 4. Discussion

In summary, dMRI-derived RD and FA in select frontal and temporal gray matter regions were associated with survival and symptom interference, with several associations moderated by the DTI-ALPS index. Higher FA in the extreme capsule was associated with longer survival, whereas lower RD in the uncinate fasciculus was associated with reduced activity-related and affective symptom interference. Entorhinal cortex FA showed hemisphere-specific associations with survival, while the relationship between caudal anterior cingulate FA and affective symptom interference varied with the DTI-ALPS index. Collectively, these findings suggest region-specific relationships between brain microstructure and DTI-ALPS index and highlight complementary dMRI-based markers of neural resilience in long-term cancer survivorship. By integrating tissue microstructure with a noninvasive MRI proxy of glymphatic function, this study highlights the potential of advanced dMRI to characterize brain health and clinically meaningful outcomes following cancer and its treatment. However, the modest and heterogeneous sample may have limited statistical power to detect smaller effects; therefore, these findings, including nonsignificant associations, should be interpreted cautiously and require confirmation in larger, longitudinal cohorts.

### 4.1. Survival, Microstructure, and Glymphatic Modulation

Our results indicate that survival in HN cancer survivors is closely linked to the microstructural integrity of select frontal, temporal, and WM pathways, with ALPS index moderating several of these relationships. Within GM, reduced RD in the left MOFC was robustly associated with longer survival. The MOFC is critical for integrating emotional and reward-related information, regulating stress responses, and guiding decision-making under uncertainty [22,53,54]. Reduced RD likely reflects preserved microstructural coherence, including maintained dendritic and axonal organization, which may support adaptive neural responses to systemic and treatment-related stressors [55,56,57,58]. Importantly, this survival benefit was enhanced in individuals with higher ALPS index, suggesting that efficient glymphatic clearance preserves frontal GM integrity, potentially facilitating more effective stress adaptation and resilience at the neural level. WM integrity also contributed uniquely to survival outcomes. Higher FA in the right extreme capsule—a tract connecting frontal and temporal regions—was robustly associated with longer survival. The extreme capsule underlies long-range fronto-temporal communication and supports functions such as language processing, executive control, and integrative cognition [37,59,60]. Preserved FA in this pathway indicates coherent and efficient connectivity, which may enable adaptive cognitive and emotional regulation, indirectly and potentially influencing survival. Together, these findings suggest that survival may be supported by the preservation of interconnected frontal–temporal networks, with ALPS index facilitating maintenance of these circuits under the neurobiological burden of cancer and its treatment.

A particularly novel observation was that associations between entorhinal cortex FA and survival were hemisphere specific. Higher FA in the left entorhinal cortex and lower FA in the right entorhinal cortex were both linked to longer survival. The entorhinal cortex serves as a central hub for memory encoding, spatial navigation, and multisensory integration, connecting the hippocampus with neocortical areas [21,61,62]. Therefore, higher FA in the left hemisphere likely reflects preserved microstructural organization supporting cognitive and mnemonic processes, maintaining the brain’s adaptive capacity and overall functional reserve. Conversely, the association of lower right-sided FA with greater survival may represent selective pruning or reorganization that enhances neural efficiency under stress [63,64,65]. This hemisphere-specific pattern highlights the complex and lateralized nature of temporal lobe contributions to survival, suggesting that left and right entorhinal cortices may confer complementary benefits—one preserving cognitive stability, the other optimizing efficiency and adaptability. Consistent with this interpretation, experimental modulation of the entorhinal cortex has demonstrated hemispheric specialization, with left-sided engagement preferentially supporting abstract and mnemonic aspects of spatial processing, while right-sided modulation enhances sensory-driven navigation and adaptive performance, underscoring complementary functional roles across hemispheres [66].

### 4.2. Symptom Interference, Microstructure, and Glymphatic Modulation

Beyond survival, microstructural integrity was associated with both activity-related and affective symptom interference, with ALPS index serving as a critical moderator. Within WM, reduced RD in the left UF was associated with lower activity-related and affective interference, particularly at lower ALPS values. This suggests that when glymphatic clearance is compromised, preserved fronto-temporal connectivity becomes especially important for supporting adaptive emotional regulation, social cognition, and coping mechanisms. The UF links orbitofrontal and anterior temporal regions, enabling integration of affective and cognitive signals critical for regulating emotional responses to stress and illness [31,67,68]. Within GM, reduced RD in the left STC and reduced FA in the right CACC were linked to lower affective interference. The STC is a hub for auditory processing, social perception, and multisensory integration, which may contribute to modulation of affective responses and interpersonal functioning [69,70,71]. The CACC, a core node of the cingulo-limbic network, supports conflict monitoring, emotional appraisal, and autonomic regulation [72,73,74,75,76]. Notably, the association between lower FA in the right CACC and lower affective interference may reflect region-specific microstructural adaptations related to symptom experience. The direction of this association was unexpected and its underlying biological significance remains unclear. Therefore, this finding should be interpreted cautiously and considered exploratory, warranting further investigation in larger, longitudinal cohorts. ALPS index moderated these relationships. For the STC, the RD–interference association remained significant at lower ALPS values, indicating that preserved GM microstructure can confer resilience only under suboptimal clearance. For the CACC, the association between FA and affective interference was also strongly moderated by ALPS index. At low ALPS, lower FA was associated with greater affective interference, indicating increased vulnerability when clearance is compromised. In contrast, when ALPS was high, lower FA was associated with lower interference, suggesting that efficient glymphatic function can buffer the functional consequences of reduced microstructural integrity. Together, these findings indicate that the impact of CACC microstructural alterations on affective interference depends on glymphatic-mediated clearance, with ALPS determining whether reduced FA reflects vulnerability or adaptive resilience.

### 4.3. Survival and Symptom Interference: An Integrative Perspective

Together, our findings demonstrate that survival and symptom interference in HN cancer survivors are governed by the dynamic interplay between GM and WM microstructural integrity and ALPS index. ALPS index may amplify the protective effects of preserved tissue, supporting long-term survival and neural resilience. In contrast, reduced clearance exposes latent vulnerabilities, making microstructural preservation especially critical for maintaining adaptive emotional regulation and minimizing symptom interference. Thus, brain clearance efficiency serves as a key determinant of whether microstructural alterations translate into resilience or functional impairment during survivorship.

Consistent with this framework, ALPS showed a negative association with RD, indicating that greater ALPS index aligns with lower radial diffusivity and better tissue integrity. In contrast, the absence of a robust positive association between ALPS and FA—aside from a trend-level effect in the left entorhinal cortex—suggests that FA reflects microstructural properties less directly influenced by clearance mechanisms. Notably, the survival benefits of preserved microstructural integrity were most pronounced under conditions of higher ALPS index. Collectively, these findings indicate that ALPS exerts outcome-specific moderating effects: the association of microstructural integrity with survival was stronger at higher ALPS (e.g., as observed for the MOFC), whereas the association of microstructural integrity with symptom interference was more pronounced at lower ALPS (e.g., as observed for the UF and CACC). Thus, the differential moderation by ALPS suggests that the relationship between structural integrity and clinical outcomes is context-dependent and may vary according to the level of ALPS index. These findings further suggest that glymphatic function may support neural resilience partly by promoting immune and metabolic homeostasis, highlighting its potential role as a mechanism linking tissue integrity, symptom burden, and survivorship outcomes.

### 4.4. Healthy Control Comparison

Although the present study did not include a healthy control group, prior dMRI literature suggests that cancer survivors may exhibit lower FA and higher RD in selected brain regions compared with healthy individuals [77,78], consistent with alterations in microstructural organization. However, the magnitude and regional distribution of these differences may vary according to cancer characteristics, treatment exposure, and other clinical factors, and a consistent pattern of hemispheric laterality is not necessarily expected. Thus, the present findings should be interpreted as associations within the HN cancer survivorship population rather than as evidence of deviation from healthy brain structure. Future studies incorporating appropriately matched healthy controls scanned using harmonized acquisition and processing protocols will be important for determining the magnitude, regional specificity, and laterality of microstructural differences associated with HN cancer survivorship.

### 4.5. Clinical and Translational Implications

This study highlights glymphatic function as a promising dual resilience mechanism, capable of both supporting survival and mitigating symptom burden in long-term HN cancer survivors. Strategies that optimize sleep, modulate vascular or cerebrospinal fluid dynamics, or enhance interstitial fluid clearance could complement approaches focused on preserving microstructural integrity. The identification of key GM regions and WM pathways—including the orbitofrontal cortex, entorhinal cortex, superior temporal cortex, caudal anterior cingulate cortex, extreme capsule and uncinate fasciculus—offer tangible targets for longitudinal monitoring and individualized rehabilitation programs, such as cognitive-emotional training or neuromodulation. Importantly, both the main and exploratory findings suggest that higher ALPS index supports structural brain integrity, promoting preserved microstructural organization in regions critical for cognitive and affective processes. By facilitating adaptive neural remodeling, protecting against microstructural decline, and preserving dendritic architecture and WM connectivity, glymphatic function may enhance resilience, reduce symptom interference, and improve functional outcomes, emphasizing its potential as a mechanistic target for survivorship interventions.

### 4.6. Limitations and Future Directions

Several limitations warrant consideration, yet they also provide context for interpreting the broader significance of our findings. First, a consideration when interpreting diffusion metrics is that FA is most commonly characterized in the context of WM, where higher FA is typically associated with greater microstructural integrity. In cortical GM, FA is influenced by additional factors such as dendritic architecture, cellular density, and extracellular fluid dynamics, meaning that lower FA may not necessarily indicate degeneration. Rather, these variations may reflect region-specific microstructural reorganization, compensatory remodeling, or adaptive plasticity. Viewed alongside glymphatic function and survivorship outcomes, these findings highlight the importance of interpreting diffusion metrics in a region-, tissue-, and hemisphere-specific manner, suggesting that deviations from canonical WM–based assumptions may reflect meaningful adaptive neurobiological processes that support survival and symptom resilience. Second, the relatively limited number of diffusion directions available in this retrospective clinical dataset (20–27 directions) is an additional methodological limitation. Although the acquisition provided sufficient diffusion information for the present ALPS-focused analyses following rigorous quality control, higher-angular-resolution and multi-shell acquisitions with a greater number of diffusion directions would be preferable for resolving complex fiber architecture and for validating tractography-based findings. Third, while the cross-sectional design precludes determination of causality or directionality, the observed associations (e.g., between microstructural characteristics and subsequent survival and between DTI-ALPS and microstructural characteristics) provide a foundation for future longitudinal studies to clarify temporal relationships and underlying mechanisms. Fourth, the predominantly male and older composition of the cohort limits the generalizability of the findings to populations with different demographic and clinical characteristics, including younger patients and women. Fifth, because DTI-ALPS provides an indirect measure of glymphatic function, future studies in more diverse populations using multimodal imaging approaches, including ASL and other CSF- and glymphatic-sensitive techniques, will be important to further validate the biological interpretation and generalizability of these findings. Sixth, although unmeasured factors such as lifestyle, systemic inflammation, and tumor type and grade may have influenced the observed associations, the consistency of the region- and tract-specific findings supports their potential relevance to compensatory and plasticity-related processes. In addition, the clinical heterogeneity of the cohort, including differences in cancer type, stage, treatment, and time since treatment, is another limitation. Detailed clinical information was not consistently available for all participants, and treatment regimens were highly heterogeneous, with several categories represented by small numbers. Including these factors as covariates would have substantially reduced statistical power and potentially yielded unstable estimates. Thus, residual confounding by cancer characteristics and treatment history cannot be excluded. Future prospective studies with larger samples should systematically collect detailed cancer and treatment characteristics, including treatment modality, treatment sequence, time since treatment, and complete survival information, including time to death and last known survival status. This will further allow conducting more appropriate survival models such as Cox proportional hazards. Larger samples would also permit adequately powered stratified and multivariable analyses to better disentangle such effects. Longitudinal assessment with MRI obtained before and at standardized intervals following treatment would further help distinguish cancer- and treatment-related changes from pre-existing or disease-related differences in brain microstructure.

## 5. Conclusions

In conclusion, this study demonstrates that dMRI-derived markers of brain microstructure and glymphatic function are associated with survival and symptom interference in long-term HN cancer survivors. We identify region-specific GM and WM microstructural markers of resilience and show that DTI-ALPS index modifies these associations. By integrating dMRI measures of tissue microstructure and glymphatic function, our findings provide a neuroimaging framework for characterizing brain health, vulnerability, and resilience in cancer survivorship. These results highlight the potential of dMRI-based biomarkers to inform the assessment of long-term neurological and functional outcomes following cancer and its treatment.

## Figures and Tables

**Table 1:** Patient characteristics.

Characteristics		Overall Group (N = 135)	Survival Groups **		
			Alive (N = 86)	Decreased (N = 46)	*p*-Value (Two Sample *t*-Test/Chi-Squared
Mean Age in years (SD) at	MRI scan	66.04 (11.83)	64.18 (13.05)	69.05 (8.63)	t(130) = 2.28, *p* = 0.02 *
	MDASI data collection	67.87 (10.94)	66.47 (11.73)	69.83 (8.92)	t(130) = 1.70, *p* = 0.09
	Last known survival status	73.63 (10.31)	73.09 (10.99)	73.94 (8.77)	t(130) = 0.45, *p* = 0.65
	Diagnosis ***	58.90 (11.59)	57.76 (12.74)	60.54 (8.95)	t(124) = 1.29, *p* = 0.20
Sex (F/M)		28/107	15/71	12/34	χ^2^(1) = 1.38, *p* = 0.24
Mean activity-related inference scores (SD)		1.41 (2.17)	1.31 (2.07)	1.68 (2.41)	t(130) = 0.94, *p* = 0.35
Mean affective inference scores (SD)		1.07 (1.88)	1.09 (2.04)	1.11 (1.62)	t(130) = 0.06, *p* = 0.95
Average time **** (SD) in years between MRI scan and MDASI data collection		1.80(4.82)	2.26 (4.95)	0.77 (4.50)	t(130) = 1.70, *p* = 0.09

* *p* < 0.05 (significant). ** Survival status was not available for 3 patients. *** Diagnosis date was not available for 6 patients. **** Positive values indicate that the MRI scan was acquired before MDASI data collection, whereas negative values indicate that the MRI scan occurred after MDASI data collection.

**Table 2:** Distribution of participants by tumor cancer type and primary tumor location (*N* = 135).

Tumor/Cancer Type	N	%
Mucosal Head and Neck Squamous Cell Carcinoma (Larynx/Glottis/Supraglottis; Oropharynx/Base of Tongue/Tonsil; Oral Cavity–Buccal, Gum, Mouth, Floor of Mouth, Tongue, Cheek; Nasopharynx/Pharynx)	74	54.8
Cutaneous Squamous Cell Carcinoma (Skin)	9	6.7
Melanoma–Primary (Face, Cheek, Scalp, Skin, Glabella, Temple, Parotid)	12	8.9
Melanoma–Metastatic (Scalp, Ear Lobe)	3	2.2
Salivary Gland–Parotid Malignancies	6	4.4
Other Tumors (Mandible, Sinus, Retromolar, Hypopharynx)	5	3.7
Personal History Codes (prior H&N cancer)	26	19.3
Total	135	100.0
Primary Tumor Location	N	%
Larynx (Glottis/Supraglottis)	15	11.1
Oropharynx (Base of Tongue/Tonsil)	28	20.7
Oral Cavity (Buccal, Gum, Mouth, Floor of Mouth, Tongue, Cheek)	21	15.6
Nasopharynx/Pharynx	10	7.4
Skin/Face/Scalp (Cutaneous SCC; Melanoma–Primary and Metastatic involving Face, Cheek, Scalp, Skin, Glabella, Temple, Ear Lobe, Parotid)	24	17.8
Parotid/Salivary Gland	6	4.4
Other Sites (Mandible, Sinus, Retromolar, Hypopharynx)	5	3.7
Personal History (historical primary site: Lip/Oral Cavity/Pharynx/Nasal Cavities/Tongue/Oropharynx) *	26	19.3
Total	135	100.0

* For participants categorized under Personal History, primary tumor site reflects historical diagnosis and may not correspond to treatment fields at the time of MRI acquisition.

**Table 3:** Regions and pathways of interest.

31 Bilateral Gray Matter Cortical and 7 Bilateral Gray Matter Subcortical Regions of Interest (Total = 76)	21 Bilateral and 3 Midline Major White Matter Pathways of Interest (Total = 45)
**Cortical Regions (Bilateral)** Caudal Anterior Cingulate Caudal Middle Frontal Cuneus Entorhinal Cortex Fusiform Inferior Parietal Inferior Temporal Isthmus Cingulate Lateral Occipital Lateral Orbitofrontal Lingual Medial Orbitofrontal Middle Temporal Parahippocampal Paracentral Pars Opercularis Pars Orbitalis Pars Triangularis Pericalcarine Postcentral Posterior Cingulate Precentral Precuneus Rostral Anterior Cingulate Rostral Middle Frontal Superior Frontal Superior Parietal Superior Temporal Supramarginal Transverse Temporal Insula **Sub-Cortical Regions (Bilateral)** Caudate Putamen Pallidum Amygdala Thalamus Hippocampus Nucleus Accumbens	**Projection Pathways (Bilateral)** Acoustic Radiation Corticospinal Tract Corticostriatal Pathway Corticothalamic Pathway Fornix Optic Radiation Frontopontine Tract Occipitopontine Tract Parietopontine Tract Temporopontine Tract **Association Pathways (Bilateral)** Arcuate Fasciculus Cingulum Extreme Capsule Frontal Aslant Tract Inferior Fronto Occipital Fasciculus Inferior Longitudinal Fasciculus Middle Longitudinal Fasciculus Superior Longitudinal Fasciculus U-fiber Uncinate Fasciculus Vertical Occipital Fasciculus **Commissural Pathways (Midline)** Anterior Commissure Corpus Callosum Posterior Commissure

**Table 4:** Diffusion measures associated with outcome measures (i.e., survival and interference): ALPS moderation (β coefficients, interaction effects, and *p*-values).

Outcome Metric	Tract/Region	Regression Analysis (β)	ALPS Moderation	Interpretation
Survival				
RD	L. MOFC ↓	−1.95 ****	**χ^2^** = 7.81, *p* = 0.005 ** (ALPS threshold ≥ −0.11, *p* ≤ 0.05)	Lower RD in L. MOFC predicts better survival, especially when ALPS is high.
FA	L. ENT ↑	9.37 ****	**χ^2^** = 0.81, *p* = 0.37 ^n.s.^	Higher FA in L. ENT predicts better survival; not moderated by ALPS.
FA	R. ENT ↓	−7.29 ****	**χ^2^** = 0.52, *p* = 0.47 ^n.s.^	Lower FA in R. ENT predicts better survival; not moderated by ALPS.
FA	R. EXT CAP ↑	5.04 ***	**χ^2^** = 1.42, *p* = 0.23 ^n.s.^	Higher FA in R. EXT CAP predicts better survival; not moderated by ALPS.
**Activity-related Interference**				
RD	L. UF ↓	6.61 ***	F (1,128) = 4.11, *p* = 0.04 ** (ALPS threshold ≤ −0.01, *p* ≤ 0.05)	Lower RD in L. UF predicts lower activity-related interference, especially when ALPS is low.
**Affective Interference**				
RD	L. STC ↓	5.82 ****	F (1,128) = 3.17, *p* = 0.08 * (ALPS threshold ≤ −0.003, *p* ≤ 0.05)	Lower RD in L. STC predicts lower affective interference, especially when ALPS is low.
FA	R. CACC ↓	16.47 ****	F (1,128) = 9.16, *p* = 0.003 ** (ALPS threshold ≥ 0.04, *p* ≤ 0.05) (ALPS threshold ≤ −0.32, *p* ≤ 0.06)	Lower FA in R. CACC predicts lower affective interference, especially when ALPS is high. Lower FA in R. CACC predicts greater affective interference, especially when ALPS is low.
RD	L. UF ↓	4.08 ***	F (1,128) = 4.47, *p* = 0.04 ** (ALPS threshold ≤ 0.003, *p* ≤ 0.05)	Lower RD in L. UF predicts lower affective interference, especially when ALPS is low.

↓ indicates “decrease” and ↑ indicates “increase”.  For 76 GM regions: **** survived multiple comparison correction (i.e., p < 0.05/76 = 0.0006). For 45 WM pathways: *** survived multiple comparison correction (i.e., p < 0.05/45 = 0.001). For ALPS: * p < 0.1 (significant at trend-level); ** p < 0.05; n.s.: non-significant (p > 0.1). Abbreviations: L. MOFC: Left Medial Orbitofrontal Cortex; L./R. ENT Cortex: Left/Right Entorhinal Cortex; R. EXT CAP: Right Extreme Capsule; L. UF: Left Uncinate Fasciculus; L. STC: Left Superior Temporal Cortex; R. CACC: Right Caudal Anterior Cingulate Cortex.

## Data Availability

The head and neck (HN) cancer survivor data were collected at The University of Texas MD Anderson Cancer Center under an IRB-approved clinical research protocol. Due to institutional policies, patient confidentiality protections, and regulatory restrictions, these data are not publicly available. De-identified data and customized analysis codes/scripts may be available from the University of Texas MD Anderson Cancer Center upon reasonable request and subject to institutional approval and execution of a data use agreement. Requests can be sent to the University of Texas MD Anderson Cancer Center via email at ClinicalResearchHelp@mdanderson.org. Data requests can also be directed to this manuscript’s corresponding author, subject to the same institutional approvals.
